# Ubiquitin specific protease 19 involved in transcriptional repression of retinoic acid receptor by stabilizing CORO2A

**DOI:** 10.18632/oncotarget.8976

**Published:** 2016-04-25

**Authors:** Key-Hwan Lim, Jong-Ho Choi, Jung-Hyun Park, Hyeon-Ju Cho, Jang-Joon Park, Eung-Ji Lee, Lan Li, Young-Kil Choi, Kwang-Hyun Baek

**Affiliations:** ^1^ Department of Biomedical Science, Bundang CHA Hospital, Bundang-Gu, Seongnam-Si, Gyeonggi-Do 463-400, Republic of Korea; ^2^ Department of Internal Medicine, College of Medicine, Bundang CHA Hospital, Bundang-Gu, Seongnam-Si, Gyeonggi-Do 463-400, Republic of Korea; ^3^ Department of Internal Medicine, CHA University, CHA General Hospital, Nonhyon-ro, Grangnam-Gu, Seoul 135-081, Republic of Korea

**Keywords:** adipogenesis, CORO2A, deubiquitinating enzyme, USP19

## Abstract

Deubiquitination via deubiquitinating enzymes (DUBs) has been emerged as one of the important post-translational modifications, resulting in the regulation of numerous target proteins. In this study, we screened new protein biomarkers for adipogenesis, and related studies showed that ubiquitin specific protease 19 (USP19) as a DUB is gradually decreased during adipogenesis and it regulates coronin 2A (CORO2A) as one of the components for the nuclear receptor co-repressor (NCoR) complex in some studies. The regulation of CORO2A through the deubiquitinating activity of USP19 affected the transcriptional repression activity of the retinoic acid receptor (RAR), suggesting that USP19 may be involved in the regulation of RAR-mediated adipogenesis.

## INTRODUCTION

Deubiquitination is the opposite process of ubiquitination and is mediated by deubiquitinating enzymes (DUBs) [1]. DUBs have the ability to detach ubiquitin molecules from ubiquitinated substrates, changing the fate of these substrates, including their activity or stabilization. Accordingly, DUBs have been thought to be pivotal mediators in diverse cellular systems including signal transduction, cell cycle regulation, cell proliferation, and cell death [2]. Thus far, approximately 100 DUBs have been identified and grouped into at least five classes, ubiquitin specific proteases (USPs), ubiquitin carboxy-terminal hydrolases (UCHs), ovarian-tumor proteases (OTUs), Machado-Joseph diseases, and JAB1/MPN/MOV34 metalloenzymes (JAMMs) [2].

USP19 is a subfamily of the USPs which has the most number of DUBs (~55 USPs). Several studies have suggested that USP19 has diverse roles in cellular processes, including cell cycle regulation, the response to hypoxia, and muscle atrophy [3–5]. The noncatalytic activity of USP19 has been described. For example, USP19 increased the stability of the cellular inhibitors of apoptosis 1 (c-IAP1) and c-IAP2 [6]. USP19 was shown to be involved in the unfolded protein response (UPR) and in the ER-associated degradation (ERAD) pathway [7]. These results indicate that USP19 can mediate a variety of mechanisms within cells.

Several studies have revealed the presence of histone deacetylase 3 (HDAC3), G-protein pathway suppressor 2 (GPS2), transducin β-like-1 (TBL1), TBL-related-1 (TBLR1), Kaiso, TAK-binding protein 2 (TAB2), and coronin 2A (CORO2A) in the nuclear co-repressor (NCoR) complex [8–12]. The NCoR co-repressor has critical functions in nuclear receptor-mediated gene repression by interacting with partners such as the thyroid hormone receptor (TR) and the retinoic acid receptor (RAR). NCoR is phosphorylated by the Akt-linked phosphorylation on Ser1450 to induce protein folding, and misfolded and degenerated NCoR are associated with acute myeloid leukemia [13]. However, the role of other post-translational modifications of co-suppressor proteins, such as deubiquitination, in the elongation of their half-life is poorly understood. Several studies have shown that THR and RAR recruit NCoR co-repressor complexes, and a domain-base approach showed that corepressor-nuclear receptor (CoRNR) box motifs ((I/L)XX(I/V)I) on the C-terminal domain of the NCoR complex are important to bind RAR [14–19]. The peroxisome proliferator-activated receptor-γ (PPAR-γ) is a well-known key factor of adipose tissue in glucose metabolism, and it has a critical role in adipocyte differentiation [20].

Coronin was first identified as a soluble actin-binding protein of *Dictyostelium discoideum* [21]. Since then, several studies have evaluated actin-related roles of coronin and found that several coronin isoforms exist. These can be classified into three different types (Types I, II, and III) by phylogenetic analysis [22]. Previous results of expression analysis with quantitative real-time PCR revealed that the highest expression of CORO2A, also known as IR10, occurred in the testis and that its expression was also relatively high in the cortex, duodenum, lymph nodes, ovaries, and uterus [23]. Although the biological functions of CORO2A are not well understood, a recent study demonstrated that it is a component of the NCoR co-repressor complex [24]. Several studies showed that the NCoR and SMRT repressed PPAR-γ gene transcription [25]. Moreover, the NCoR co-repressor was associated with the phosphorylation of PPAR-γ in adipocyte differentiation, and knock-down of the NCoR complex promoted adipogenesis [25]. Adipogenesis studies have been accessed to obesity research. Here, we screened the adipogenesis marker proteins in molecular mechanism studies. The results suggest that USP19 may be associated with the transcriptional regulation of RAR via CORO2A as one of the components for the NCoR complex during the adipogenesis.

## RESULTS

### Expression analysis of *Dubs* in adipocyte differentiation

**Figure 1:**
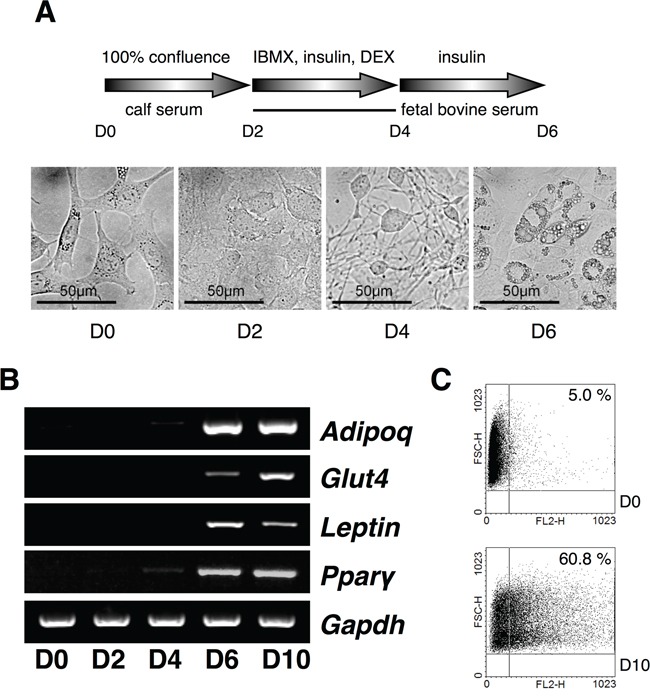
Expression analysis of *USPs* in adipocyte differentiation **A.** A scheme for the induction of adipocytes with IBMX, DEX, and insulin from 3T3-L1 cells, and the cell morphology was checked every a couple of days at original magnification 40×. **B.** Primers for *Adipoq, Glut4, Leptin*, and *Ppar-γ* were used for RT-PCR using cDNA from each phase of the differentiating adipocytes. **C.** The differentiated adipocytes were assorted and analyzed by fluorescence-activated cell sorting (FACS).

**Figure 2:**
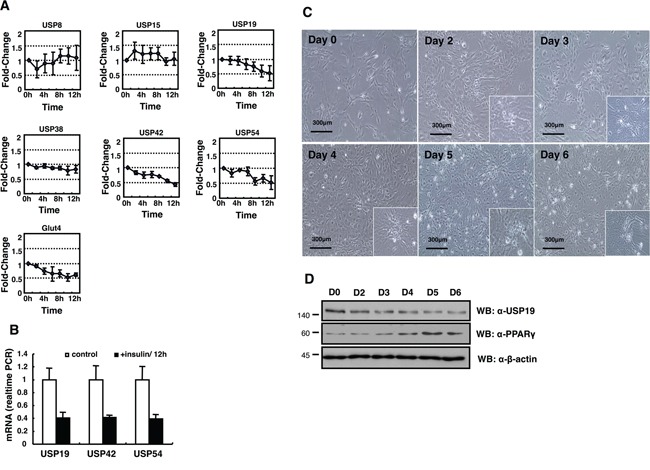
Expression profiling of *DUB* genes in the insulin-treated 3T3-L1 cells **A.** *USP8, USP15, USP19, USP42*, and *USP54* mRNA expressions were measured by real-time PCR as indicated. **B.** All data are performed three independent experiments with each insulin treated 3T3-L1 cells, and represent a means ± s.e.m. **C.** Primary MEFs induced adipocytes with IBMX, DEX, and insulin. Cell morphology was examined by a microscopy with 10× magnification. The scale bar represents 300 μm. **D.** Cell lysates were obtained from MEFs as indicated days, and analyzed by immunoblotting with an anti-USP19, an anti-PPAR-γ, and an anti-β-actin antibody.

Since the control of DUBs in adipogenesis is unknown yet, we screened *USPs* during adipogenesis using a PCR-based approach. To identify the differential expression pattern of 55 USPs and Cyld during adipocyte differentiation, insulin-treated 3T3-L1 cells were used for RT-PCR (Figure 1 and Table 1). The induction of adipogenesis by insulin resulted in significant increase for the expression of *AdipoQ, Glut4, Leptin*, and *Ppar-γ* as adipocyte-specific markers time dependently (Figure 1A-1C). Moreover, we found up-regulated and down-regulated *USPs* in differentiated adipocytes (Supplementary Data S1). We next performed a real-time PCR-based assay to estimate and confirm the expression of *USPs* in a time dependent manner after insulin treatment during adipogenesis. The results indicate that the expression of *USP19, USP42*, and *USP54* mRNA was significantly changed (Figure 2A and 2B). These findings suggest that the transcription levels of *USP19, USP42*, and *USP54* were changed during adipogenesis.

**Table 1:** A list of primers for DUB screening

Gene name	Direction	Primer sequence	Size of PCR product (bp)
USP1	Forward	5′-GGA CTG AAT AAT CTC GGC-3′	289
	Reverse	5′-GCT GAG TGG CAA GTT CAT-3′	
USP2	Forward	5′-TCT TCG TCA GCT GGT GCT-3′	293
	Reverse	5′-ATA GGA GGA CGG GGT GTA-3′	
USP3	Forward	5′-CCT TGG GCT TGT TTG ACT-3′	284
	Reverse	5′-TTA TGC CTG TCA GCT GTG-3′	
USP4	Forward	5′-AAG CAC TGC AAA GTC GAG-3′	255
	Reverse	5′-TAG CAC CTG ACC CTG GTA-3′	
USP5	Forward	5′-GTC CAC AAA GAC GAG TGC-3′	257
	Reverse	5′-CTA AGG TCA AAT CCG CCT-3′	
USP6	Forward	5′-GTT GGA ATC AAC AGC AGC-3′	266
	Reverse	5′-TAT CTT CCG GGG TTT TTC A-3′	
USP7	Forward	5′-GGG ATG GCA AAT GGT GTA-3′	300
	Reverse	5′-TCC TCT GCG ACT ATC TGC-3′	
USP8	Forward	5′-ATT TCA AGC AAC AGC AGG A-3′	263
	Reverse	5′-GGG TTT TGT CTT TGC AAT C-3′	
USP9X	Forward	5′-TAG GCT TCA AGG TTC CAG-3′	264
	Reverse	5′-CTG TGG CTG ATG AAG ACT-3′	
USP9Y	Forward	5′-CTT ATG GAT GAG GCT GTG-3′	250
	Reverse	5′-CCA CTA GCC AAC CTT TTG-3′	
USP10	Forward	5′-TTC AAG CAC ACT GAA CCC-3′	250
	Reverse	5′-TGG CAT GGC CAT TGA CCA-3′	
USP11	Forward	5′-AAA GAT GGC ACT TGG CCC-3′	326
	Reverse	5′-CCA ACC TTG TTC TTG AAC A-3′	
USP12	Forward	5′-AGT CTC CAA ATT CGC CTC-3′	261
	Reverse	5′-GTG GCT ATG CTA TGG AAG-3′	
USP13	Forward	5′-GAG TCA GGA TTC CTC CAA-3′	259
	Reverse	5′-TTG GCC AAA TGA GGA TCC-3′	
USP14	Forward	5′-GAT GAA CCT CCA ATG GTA T-3′	194
	Reverse	5′-GGC ACA GAA CCA ATA CAC-3′	
USP15	Forward	5′-AAA CCT CGC TCC GGA AAG-3′	252
	Reverse	5′-CCC TGT TCA ACC ACC TTT-3′	
USP16	Forward	5′-CAT GGG AAA GAA ACG GAC-3′	252
	Reverse	5′-CTC CTG AGA ATT CCT GCC-3′	
USP17	Forward	5′-CAA GGA GAG CTC AAG AGA-3′	259
	Reverse	5′-AAG AGA GGT TTA GCA GGG-3′	
USP18	Forward	5′-CCT GGA AGT GAA GTC GTG-3′	280
	Reverse	5′-CAA GGA GTT AAG GCA GCA-3′	
USP19	Forward	5′-TGT GGG CTA CTG CAA CCA-3′	296
	Reverse	5′-GCT GAA TGG GGT CTC TCT-3′	
USP20	Forward	5′-CCT ATT GCT GTG GCT GAT-3′	318
	Reverse	5′-GGC ATA GCC TCG GAG CAT-3′	
USP21	Forward	5′-AGT GGG ATC CAA GCT ACC-3′	279
	Reverse	5′-CTC ACA GAC TTG GAA CGG-3′	
USP22	Forward	5′-CAG CTT CAA GGT GGA CAA-3′	265
	Reverse	5′-AGA TGT AGT CCT GGC ACA-3′	
USP24	Forward	5′-GGC TGG ATA ACT TTG AAC T-3′	209
	Reverse	5′-ACT TTG GAT GAA AGT CCT G-3′	
USP25	Forward	5′-TAT CTA GAG CAG CCA TCA A-3′	230
	Reverse	5′-GCC TGG TTC TGG ATA AAG-3′	
USP26	Forward	5′-GTC CAG ATG TGG AGT GCA-3′	240
	Reverse	5′-GCC GAA TAC TAC CTT GAG-3′	
USP27	Forward	5′-CCA CTC TTG CCT TTC CTG-3′	347
	Reverse	5′-CCG ATC GTA AAG CTG GAG-3′	
USP28	Forward	5′-CCC ACC TCT CAC AGT GAT-3′	314
	Reverse	5′-TAC ACA GAC ACT TTT CGG A-3′	
USP29	Forward	5′-TTG GGA AAC ACC TGT TAC T-3′	288
	Reverse	5′-TTT CAG CTG GTC TAA ACA C-3′	
USP30	Forward	5′-CAG CGC TTC CTG CGG AC-3′	266
	Reverse	5′-TCC CTG GAG TAC TGG GAG-3′	
USP31	Forward	5′-ACT GGG TGA GCC GGC T-3′	268
	Reverse	5′-GAG CGC ATC TGC AGC TTA-3′	
USP32	Forward	5′-GTC CCA GAT ACA CTC AGG-3′	194
	Reverse	5′-AAT GTG TGA CTC CAG CCA-3′	
USP33	Forward	5′-GGA AGA GCA GCG AAG AG-3′	234
	Reverse	5′-CCC AAA CGT TCT GAG GCA-3′	
USP34	Forward	5′-AAG ACA CAT CTG GAA GCG-3′	275
	Reverse	5′-CCA AAC TCC TGA AGC TGA-3′	
USP35	Forward	5′-TGT TCG CAG TCA TCT CCT-3′	244
	Reverse	5′-TTC TTA ACA GCA GCC AGG-3′	
USP36	Forward	5′-AAG GAC TCG GCT GAT GAT-3′	273
	Reverse	5′-GGG GAA AAG CAC TTT CTG-3′	
USP37	Forward	5′-ACT GGA GGA ATT CCA AGG-3′	287
	Reverse	5′-TAA GAA AGC TGC CTG CTG-3′	
USP38	Forward	5′-CCT TGT GCA GCA TAT TCC-3′	285
	Reverse	5′-AGA ACT GCA AGA GCA CCA-3′	
USP39	Forward	5′-CAA GTA CTT TCA AGG CCG-3′	236
	Reverse	5′-TGG TAC CAT CAT ATG CCC-3′	
USP40	Forward	5′-TGG AAT GGG GTG GAG GTT-3′	300
	Reverse	5′-GCT TCC ATT TCT GAC CCT-3′	
USP41	Forward	5′-CCT TAC GCC AGT GAC TAT-3′	250
	Reverse	5′-CAA GGA GGT AAG GCA GCA-3′	
USP42	Forward	5′-TAG CAA TGG CCT CTG GTA-3′	298
	Reverse	5′-TGG CGT GTC TTT CAA TGG-3′	
USP43	Forward	5′-CAG AAG CGG AAC AGC ATC-3′	259
	Reverse	5′-TGC CTT CAT GCT AAT GCT T-3′	
USP44	Forward	5′-ACC GAG TCC ATT TGG GCT-3′	278
	Reverse	5′-ACT TCA GGT CTC CAG TTG-3′	
USP45	Forward	5′-CGG GTG AAA GAT CCA ACT-3′	264
	Reverse	5′-ACA CTT GAG GCA CAA CCA-3′	
USP46	Forward	5′-GGA TGA GGG TAA AAA AGC T-3′	199
	Reverse	5′-CTT TCA CAG TGA ACG ACC-3′	
USP47	Forward	5′-TGT TGA AAG CTC CGA GAC-3′	278
	Reverse	5′-CTG CTG TTG TG CAG TGA −3′	
USP48	Forward	5′-CCG AAT TGC TTG GTT GGT −3′	300
	Reverse	5′-CAA GTA CTG GAG ATG CTC-3′	
USP49	Forward	5′-GAT GCA AAC ATG TAG GGC-3′	256
	Reverse	5′-TCA TTG AGC ACG TAG TCC-3′	
USP50	Forward	5′-GTG CTT CAT TGA CAT GGC-3′	258
	Reverse	5′-CTC ACT GCA GTC CTT CTT-3′	
USP51	Forward	5′-ACC CCA GAG ACT AGG AAA-3′	243
	Reverse	5′-TTC TTT GGC AAT CTG TTC TA-3′	
USP52	Forward	5′-TCT GGC AAG GTT TCC CTG-3′	187
	Reverse	5′-CAC GCA TCA TGC GCA AAT-3′	
USP53	Forward	5′-GAT ATG ACA CAG ACA GCA G-3′	299
	Reverse	5′-AAG GGA ACT TCT GCT TCC-3′	
USP54	Forward	5′-GGT AGT GTA CAA GGG ATG TT-3′	260
	Reverse	5′-GAG AGT GTC AGA TGG AAG C-3′	
USP55	Forward	5′-GCA ACC TCA TGC AGT TCT-3′	240
	Reverse	5′-AAA CCT TGA CCA CGA CCT-3′	
CYLD	Forward	5′-GCA ACC TCA TGC AGT TCT-3′	300
	Reverse	5′-AAA CCT TGA CCA CGA CCT-3′	

### CORO2A is a novel binding partner for USP19

**Figure 3:**
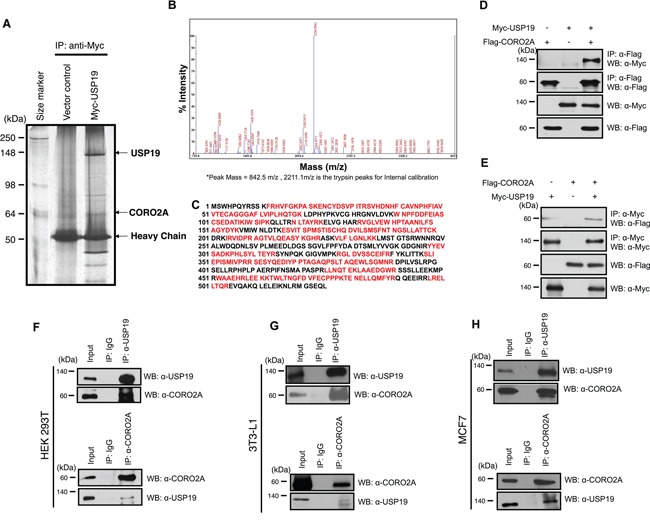
Putative binding proteins of USP19 Immunoprecipitation and MALDI-TOF-MS analyses were performed to investigate novel substrates for USP19. **A.** USP19 overexpression or a control sample in 293T cells, which were immunoprecipitated with an anti-Myc antibody and stained with the silver staining method. **B.** and **C.** Bands showing differential expression were selected and analyzed by MALDI-TOF-MS analysis. **D.** The interaction between USP19 and CORO2A was confirmed by an immunoprecipitation assay with an anti-Myc antibody and immunoblotting with anti-Flag and anti-Myc antibodies using Myc-tagged USP19 and/or Flag-tagged CORO2A overexpressed 293T cell lysates. **E.** Reciprocal data for D were obtained with respective antibodies. **F-H.** 293T, 3T3-L1, and MCF7 cell lysates were precipitated by an anti-USP19 antibody. USP19 and CORO2A were detected by anti-USP19 and anti-CORO2A antibodies, respectively. F-H, 293T, 3T3-L1, and MCF7 cell lysates were precipitated by an anti-CORO2A antibody. CORO2A and USP19 were detected by indicated antibodies.

The expression of *USP19* was most significantly suppressed in adipocyte differentiation (Figure 2). In addition, we monitored the expression of USP19 during adipogenesis processing with primary mouse embryo fibroblasts (MEFs) to confirm previous results (Figure 2A and 2B). While adipocytes were differentiated, the expression level of USP19 was decreased (Figure 2C and 2D) and the expression of PPAR-γ as a marker protein for adipogenesis was increased. To gain insights into USP19 function in adipogenesis, we performed immunoprecipitation and MALDI-TOF-MS analyses to identify the binding partners of USP19. Purified binding proteins from Myc-tagged USP19-overexpressed 293T cells were separated with SDS-PAGE followed by silver staining and mass spectrometry (Figure 3A). The result of the mass spectrometry analysis of differentially appearing protein band revealed the score values, molecular weights, and partial amino acid sequences for CORO2A (Figure 3B and 3C). The results suggest that CORO2A is an USP19 binding protein (Figure 3B and 3C). We next validated the association between USP19 and CORO2A, and the regulation of CORO2A by USP19. The 293T cells were transfected with Flag-tagged CORO2A and Myc-tagged USP19. Co-immunoprecipitation assay revealed that USP19 strongly binds with CORO2A (Figure 3D and 3E). We next evaluated the endogenous binding between CORO2A and USP19. As expected, CORO2A was detected by immunoprecipitation using an anti-USP19 antibody, and reciprocal immunoprecipitation with an anti-CORO2A antibody also brought down USP19 in non-cancer cells (293T and 3T3-L1 cells) and cancer cells (MCF7 cells) (Figure 3F–3H). These results show that the binding of CORO2A and USP19 was not dependent on both normal and cancer cells. Furthermore, USP19 can be a DUB for increasing the stability of CORO2A.

### USP19 is associated with the ubiquitination of CORO2A

**Figure 4:**
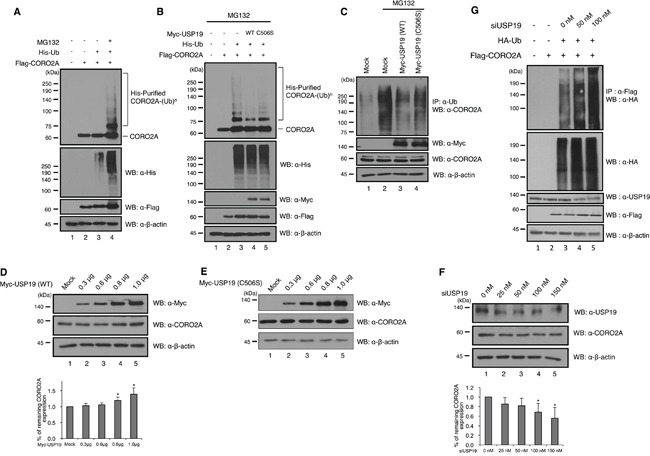
Deubiquitinating activity of USP19 on CORO2A **A.** and **B.** Cell lysates from HEK 293T cells which transfected with His-tagged ubiquitin, Flag-tagged CORO2A and/or Myc-tagged USP19 and the catalytic mutant USP19 (C506S) were subjected to *in vitro* ubiquitination and deubiquitination assay with Ni-NTA beads. MG132 (2.5μM) as a proteasome inhibitor was treated for 6 h before cell harvest. Western blotting was performed with indicated antibodies. A, The ubiquitination level of CORO2A was increased when the cells were treated with MG132 (lane 4), a proteasome inhibitor. B, The overexpression of USP19, but not the catalytic mutant USP19 (C506S), dramatically reduced the ubiquitination level of CORO2A (lanes 4 and 5). **C.** *In vivo* ubiquitination and deubiquitination assays were performed to identify the specific deubiquitinating activity of USP19 toward CORO2A. Myc-tagged USP19 and the catalytic mutant USP19 (C506S) were overexpressed in the 293T cells, and the cell lysates were used for immunoprecipitation with an anti-ubiquitin antibody. Lane 1 shows the ubiquitination of CORO2A. **D.** and **E.** USP19 and the catalytic mutant USP19 (C506S) were transfected into 293T cells by dose dependent manner (0, 0.3, 0.6, 0.8, and 1.0 μg) and cell lysates were analyzed with indicated antibodies. **F.** *USP19* siRNA was transfected into 293T cells by dose dependent manner (0, 25, 50, 100, and 150 nM), and the expression level of USP19 and CORO2A was detected by anti-USP19 and anti-CORO2A antibodies. D and F, Statistical data are presented as a means (n=3, *p<0.05). **G.** Lysates from cells which respectively transfected with *USP19* siRNA, HA-tagged *Ubiquitin*, and Flag-tagged *CORO2A* were subjected to the ubiquitination assay.

Ubiquitination of CORO2A has not been reported yet, and we confirmed that CORO2A is involved in the ubiquitin-proteasome pathway by treating the cells with MG132, as a proteasome inhibitor (Figure 4A, lane 4). Upon the overexpression of USP19, the ubiquitination level of CORO2A was significantly decreased both *in vivo* and *in vitro* conditions (Figure 4B, lane 4 and 4C, lane 3). However, the catalytic mutant USP19 (C506S) did not significantly decrease the ubiquitination level of CORO2A (Figure 4B, lane 5 and 4C, lane 4). These results indicate that USP19 may prevent proteasomal degradation of CORO2A. Since USP19 reduced the ubiquitination level of CORO2A, we next tested the expression level of CORO2A upon USP19 dose dependent expression (Figure 4D). The result showed that CORO2A was gradually increased by USP19 expression (Figure 4D). However, a catalytic mutant USP19 (C506S) did not affect the level of CORO2A (Figure 4E). Furthermore, the expression level of CORO2A was decreased with the inhibition of USP19 expression (Figure 4F). In addition, depletion of USP19 increased the level of ubiquitination for CORO2A (Figure 4G). Collectively, these data demonstrate that CORO2A is a binding protein for USP19 and USP19 stabilizes CORO2A through its deubiquitinating activity.

### USP19 and CORO2A mediate the transcriptional repression of RAR

**Figure 5:**
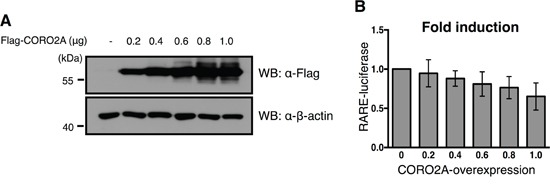
Effect of overexpression of CORO2A and USP19 on the transcriptional repression of RAR targeting elements **A.** To confirm the dose dependent increase in Flag-tagged CORO2A in the MCF7 cells that contained the RARE reporter gene, immunoblotting with the same samples was performed with anti-Flag and anti-β-actin antibodies. **B.** The luciferase activity of RAR was gradually decreased in a dose dependent manner in response to a gradual increase in Flag-tagged CORO2A in the MCF7 cells. The average of six experiments is presented, and error bars denote the standard error mean (± s.e.m.).

**Figure 6:**
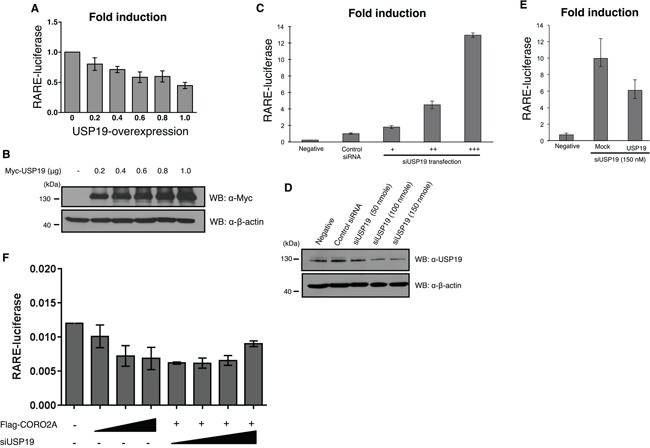
Effect of depletion of USP19 on RAR and PPAR-γ transcriptional activity **A.** and **B.** The luciferase activity of RAR was gradually decreased in a dose dependent manner with a gradual increase in Myc-tagged USP19 in the MCF7 cells that contained the RARE reporter gene. A, MCF7 cells were transfected with Myc-tagged USP19 in a dose dependent manner and the luciferase activity of RAR was gradually decreased by the expression level of USP19. B, To confirm the dose dependent increase of USP19, immunoblotting with the same samples was performed with anti-Myc and anti-β-actin antibodies. **C.** The luciferase activity of RAR was analyzed in MCF7 cells transfected with *USP19* siRNA in a dose dependent manner (+: 50 nmol, ++: 100 nmol, +++: 150 nmol), and the results indicate that the transcription level of RAR was dramatically increased. The luciferase activity of RARE was analyzed in *USP19* siRNA transfected cells. **D.** To confirm the dose dependent decrease of USP19, immunoblotting with the same samples was performed with anti-USP19 and anti-β-actin antibodies. **E.** The luciferase activity of RAR was detected in MCF7 cells co-transfected with 150 nM of *USP19* siRNA and 0.8 μg of Myc-tagged USP19. **F.** The luciferase activity of RAR in the presence or absence of CORO2A and USP19. All data obtained from six independent experiments and represent a means ± s.e.m.

Previous studies have suggested that the co-repressor NCoR complex functions in RARE transcription through DNA-binding sites for RARs [26]. Therefore, we hypothesized that USP19, which was found to bind with CORO2A, may mediate the function of the RARs via transcriptional repression of RARE. We performed luciferase assays to investigate transcriptional levels of RARE upon USP19 expression. When CORO2A was dose-dependently expressed in MCF7 cells (Figure 5A), the luciferase activity of RARE was decreased in response to a gradual increase in the level of CORO2A as expected (Figure 5B). Our study showed that USP19 has deubiquitinating activity for CORO2A (Figure 4) when we hypothesized that the expression of USP19 also affects the transcription level of RARE. Interestingly, increasing expression of USP19 also decreased approximately twenty percent of the luciferase activity of RARE (Figure 6A and 6B). On the other hand, the knock-down of USP19 mediated by the treatment of siRNA specific for *USP19* resulted in a substantial increase in RARE luciferase activity (Figure 6C and 6D). To elucidate an off-target effect by *USP19* siRNA, Myc-tagged USP19 was used for the rescue expression against *USP19* siRNA transfection, and the expression of Myc-tagged USP19 reduced the luciferase activity of RARE in USP19 depleted cells (Figure 6E). RAR target genes were decreased by the overexpression of CORO2A, and increased by the knockdown of USP19 (Supplementary Data S2). We next tested whether the CORO2A inhibits RARE luciferase activity in the USP19-defected condition (Figure 6F). Interestingly, depletion of USP19 by *USP19* siRNA with the overexpression of CORO2A did not increase the luciferase activity of RARE compared with *USP19* siRNA alone (Figure 6F). These results indicate that USP19 is associated with the regulation of transcriptional repression of RAR through CORO2A.

## DISCUSSION

Several stimulation factors such as insulin, transforming growth factor-beta (TGF-β), fibroblast growth factor (FGF), and bone morphogenetic proteins (BPMs) are required for gene expression during adipogenesis. The manner in which DUBs coordinate and regulate adipocyte gene expression or protein degradation remains unclear. In the present study, we investigated specific adipocyte differentiation marker proteins. During adipogenesis, the mRNA levels of *USP19, USP42*, and *USP54* were significantly changed (Figure 1 and Figure 2). To investigate the molecular mechanisms of USP19 in the deubiquitination system during adipogenesis, the binding substrates of USP19 were screened (Figure 3 and Figure 4). CORO2A, an NCoR complex protein, was isolated with an anti-USP19 by immunoprecipitation. And USP19 deubiquitinated CORO2A (Figure 4), suggesting that USP19 might have function to stabilize NCoR co-repressor complex in cells.

CORO2A was identified as a component of the NCoR complex, and it has been suggested that CORO2A has a role in transcriptional repression of its target genes through the regulation of NCoR turnover [27]. NCoR co-repressor complexes are known to play a role in the transcriptional repression of target genes via nuclear receptors, including RAR and THR, and other molecules including Sin3, HDAC1, HDAC3, TBL1/R1, GPS2, Kaiso, JMJD2A, and CORO2A [8, 10, 12, 28–31]. RAR and retinoid X receptor (RXR), act as transcription factors encoded by genes containing RAR response elements (RARE) or RXR response elements (RXRE) [32]. Their binding influences the transcriptional repression of RAR, which is a binding partner of the NCoR complex [33]. For example, the transcriptional activation of RAR target genes was increased upon depletion of HDAC3 but not NCoR in RAR-expressing cells [33]. Overexpression of USP19 inhibited RARE transcription and knock-down of *USP19* using siRNA showed dramatically increased transcription of RARE (Figure 5 and Figure 6), suggesting that USP19 increases the stability of CORO2A by DUB activity, and that binding of these two protein may be associated with the function of NCoR. Together, our results demonstrate the importance of the interaction between USP19 and CORO2A in the regulation of RAR, which is possibly involved in adipogenesis. Tip60, which is a factor for adipocyte differentiation, was regulated by USP7, and their binding was associated with adipogenesis [37]. Fatty acid synthase (FAS), a regulator for adipocyte differentiation, is also stabilized by USP2 [38]. Clinically, the modulation of retinoic acid receptors (RARs) by NCoR is regarded as a potential target for development of metabolic diseases and anti-cancer drugs. Up to date, only few studies were performed for adipogenesis-related DUBs. Based on our results, we screened several USPs that may have important roles in adipocyte differentiation. Our molecular mechanism study revealed that USP19 has a role as a co-repressor in the transcriptional repression of RAR via the stabilization of CORO2A by its DUB activity, and suggests the possibility that USP19 may be a potent target for anti-cancer and metabolic diseases.

## MATERIALS AND METHODS

### Bioinformatics and LC-MS/MS analysis

Conserved domain predictions were analyzed with the rpsblast program at http://www.ncbi.nlm.nih.gov/Structure/cdd/wrpsb.cgi. Other programs including Superfamily (http://supfam.mrc-lmb.cam.ac.uk/SUPERFAMILY/), SMART (http://smart.embl-heidelberg.de/), Pfam (http://www.sanger.ac.uk/Software/Pfam/), InterPro (http://www.ebi.ac.uk/interpro/), and Prosite (http://us.expasy.org/prosite/) were also used. Alignment was performed with the DNASTAR program and MultiAlign (http://prodes.toulouse.inra.fr/multalin/). After MALDI-TOF-MS analysis, proteins were identified from peptide mass maps with MASCOT (http://www.matrixscience.com) and MS-Fit (http://prospector.ucsf.edu) using monoisotopic peaks. Agilent 1100 Series nano-LC and LTQ- mass spectrometer (Thermo Electron, Bremen, Germany) were used for LC–MS/MS analysis as previously described [39]. The capillary column was packed with the Magic C18 stationary phase (5 μm particle, 100 Å pore size) (Michrom Bioresources, Auburn, CA, USA). The mobile phases A and B for the LC separation were performed with 0.1% formic acid in deionized water or acetonitrile, respectively. The chromatography gradient program, flow rate, and MS/MS scan were performed as previously described [39]. Identification of peptide sequence was performed with SEQUEST software (Thermoquest, San Jose, CA, USA).

### Plasmid and siRNA

The KIAA0891 clone containing the coding region of human *USP19* was kindly donated by Dr. Nagase at the KAZUSA Institute in Japan [40]. The coding region of human *USP19* was subcloned into pcDNA3-Myc (Invitrogen, Carlsbad, CA, USA). Myc-tagged USP19 (C506S) mutant was generated by site-directed mutagenesis. Using a commercial clone (IMAGE clone: 3350035) containing the coding region of *Coro2A*, we subcloned *Coro2A* into a pCS4-Flag vector (Invitrogen, Carlsbad, CA, USA). His-tagged ubiquitin was cloned as previously described [41]. Different concentrations of *USP19* siRNA (0.5, 1 and 1.5 nmol) were transfected into cells by using an Opti-MEM and RNAimax (Invitrogen, Carlsbad, CA, USA) mixture according to the manufacturer's instructions. The *USP19* sense siRNA sequences were; 5′-GGA GGA GAU GGC AGU GGC A-3′. The anti-sense siRNA sequences were; 5′-UGC CAC UGC CAU CUC CUC C-3′ (UbiProtein Corp, Seongnam, Korea).

### Cell culture and transfection

Human embryonic kidney 293T cells, 3T3-L1 preadipocytes, and MCF7 breast cancer cells were cultured in Dulbecco's modified Eagle's medium (DMEM, GIBCO, Rockville, MD, USA) supplemented with 10% fetal bovine serum (GIBCO, Rockville, MD, USA) and 1% penicillin/streptomycin (GIBCO Rockville, MD, USA). Mouse embryo fibroblasts (MEFs) were collected from mouse embryos at day 12.5 [42]. To induce adipocytes from 3T3-L1 cells, they were treated with 0.5 mM of 3-isobutyl-1-methylxanthine (IBMX), 1 μM of dexamethason (DEX) and 10 μg/ml of insulin. The cells were grown in the 5% CO_2_ incubator. Transfection of the 293T cells was performed by 10 mM polyethlylenimine (PEI, Polysciences, Warrington, PA, USA).

### RNA isolation and quantitative real-time PCR

The total RNA was isolated from the 3T3-L1 and MCF7 cells using TRIzol according to the manufacturer's protocol (Invitrogen, Carlsbad, CA, USA). RT-PCR and quantitative real-time PCR were performed with an SYBR green PCR kit (Applied Biosystems, Foster City, CA, USA).

### Silver staining, immunoprecipitation, immunoblotting, and ubiquitination assay

For silver staining, a pcDNA3-Myc empty vector or pcDNA3-Myc-*USP19* was transfected into the 293T cells. After 48 hrs, the cells were harvested and lysed using a lysis buffer (50 mM Tris-HCl [pH 7.8], 150 mM NaCl, 1% Triton X-100) containing a protease inhibitor cocktail (Roche Diagnostics, Mannheim, Germany) and phenylmethylsulfonyl fluoride (PMSF) (Sigma-Aldrich, St. Louis, MO, USA). Cell lysates were immunoprecipitated with an anti-Myc antibody (9E10) for 4 hrs. After incubation with protein A/G beads (Santa Cruz Biotechnology, Santa Cruz, CA, USA) for 1 hr, beads were washed three times with a washing buffer. Silver staining was performed using a silver stain plus kit (Bio-Rad, Hercules, CA, USA) according to the manufacturer's instruction. Briefly, gels were fixed with a fixing solution containing methanol and acetic acid. After washing with distilled water two times, gels were stained with staining solutions. Finally, a stop solution containing acetic acid was used. For *in vivo* binding between CORO2A and USP19, 293T cells were transfected with pcDNA3-Myc-*USP19* and/or pCS4-Flag-*Coro2a* and/or pcDNA3-HA-*Ubiquitin*. For immunoprecipitation assay, the cell lysates were incubated with indicated antibodies for 4 hrs at 4°C. Then protein A/G PLUS agarose bead (Santa Cruz Biotechnology, Santa Cruz, CA, USA) was added and rotated for 1 hr. His-tagged ubiquitin was purified as previously described [41]. Proteins from cell lysates and immunoprecipitates were loaded into polyacrylamide gels and transferred to a polyvinylidene fluoride (PVDF) membrane (Millipore, Billerica, MA, USA). Following antibodies were used: anti-Myc, anti-CORO2A (Santa Cruz Biotechnology, Santa Cruz, CA, USA), anti-HA (12CA5), anti-Flag (Sigma-Aldrich, St. Louis, MO, USA), and anti-β-actin (Santa Cruz Biotechnology, Santa Cruz, CA, USA). Western blotting images were analyzed with Image J program.

### Antibody production

Polyclonal USP19 antisera were generated in rabbit that were immunized with a USP19 C-terminal peptide (ASRIWQELEAEEEPVPEGSGP) (UbiProtein Corp, Seongnam, Korea). Rabbit polyclonal anti-USP19 antibody was affinity-purified with rProtein A agarose column (UbiProtein Corp, Seongnam, Korea) and eluted with an elution buffer (1 M Tris [pH 9.0] and 100 mM citric acid [pH 3.0]).

### Luciferase assay

pcDNA3-Myc-*USP19*, siRNA specific for *USP19* with RARE, and PPAE luciferase vectors (Cignal Report; Qiagen, Valencia, CA, USA) were transfected into MCF7 cells positively expressing RARs. After 48 h, the transfected cells were harvested with a passive lysis buffer and incubated with a luciferase assay buffer (Promega, Madison, WI, USA). Luciferase activity for each cell lysate under different conditions was detected with a luminometer (Tecan, San Jose, CA, USA) according to manufacturer's instruction.

## SUPPLEMENTARY FIGURES


