# Supplementary material for: Ubiquitin specific protease 19 involved in transcriptional repression of retinoic acid receptor by stabilizing CORO2A
Source: Oncotarget. 2016 Apr 25;7(23):34759–72. doi: 10.18632/oncotarget.8976 (PMC5085187; doi:10.18632/oncotarget.8976)
Supplement: Supplementary file 1 [file oncotarget-07-34759-s001.pdf]

SUPPLEMENTARY FIGURES

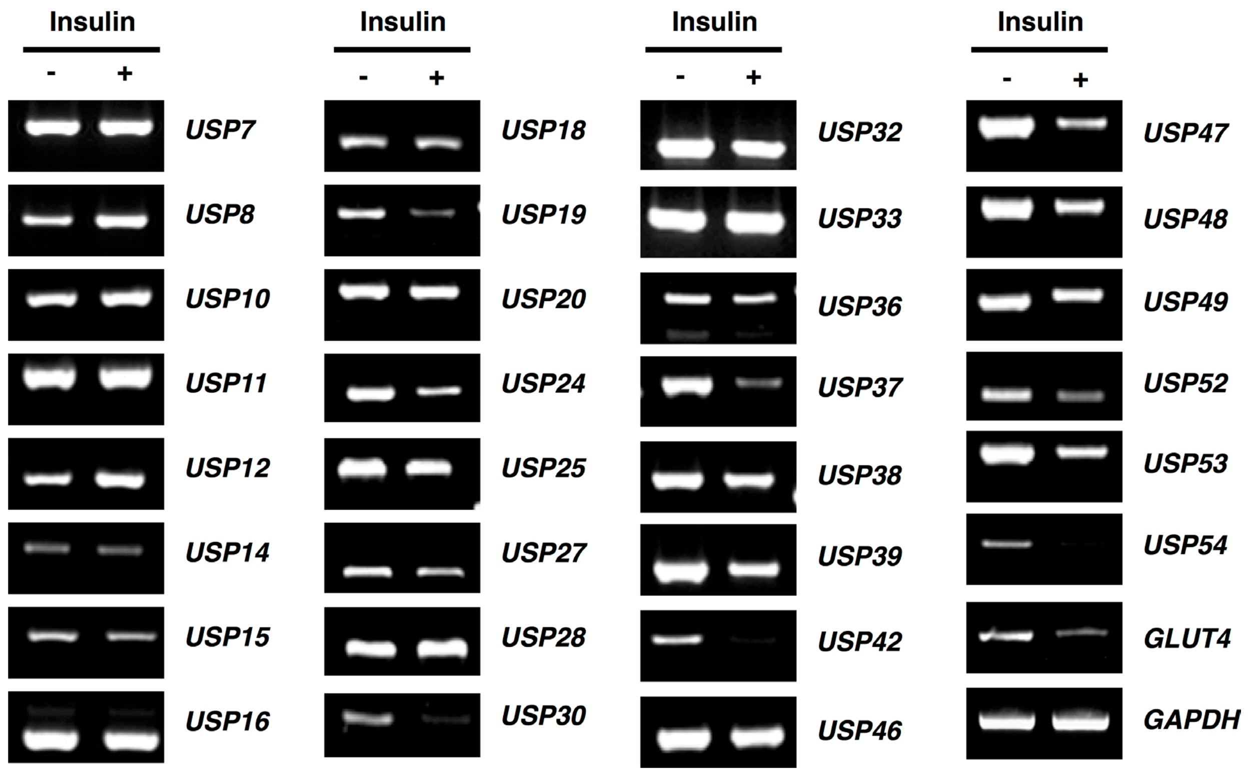

Supplementary Figure S1: Expression profiling of *Dub* genes with insulin treated 3T3-L1 cells. 3T3-L1 cells were harvested after insulin treatment, and RT-PCR was performed with cDNA from harvested cells and indicated *Dub* primers.

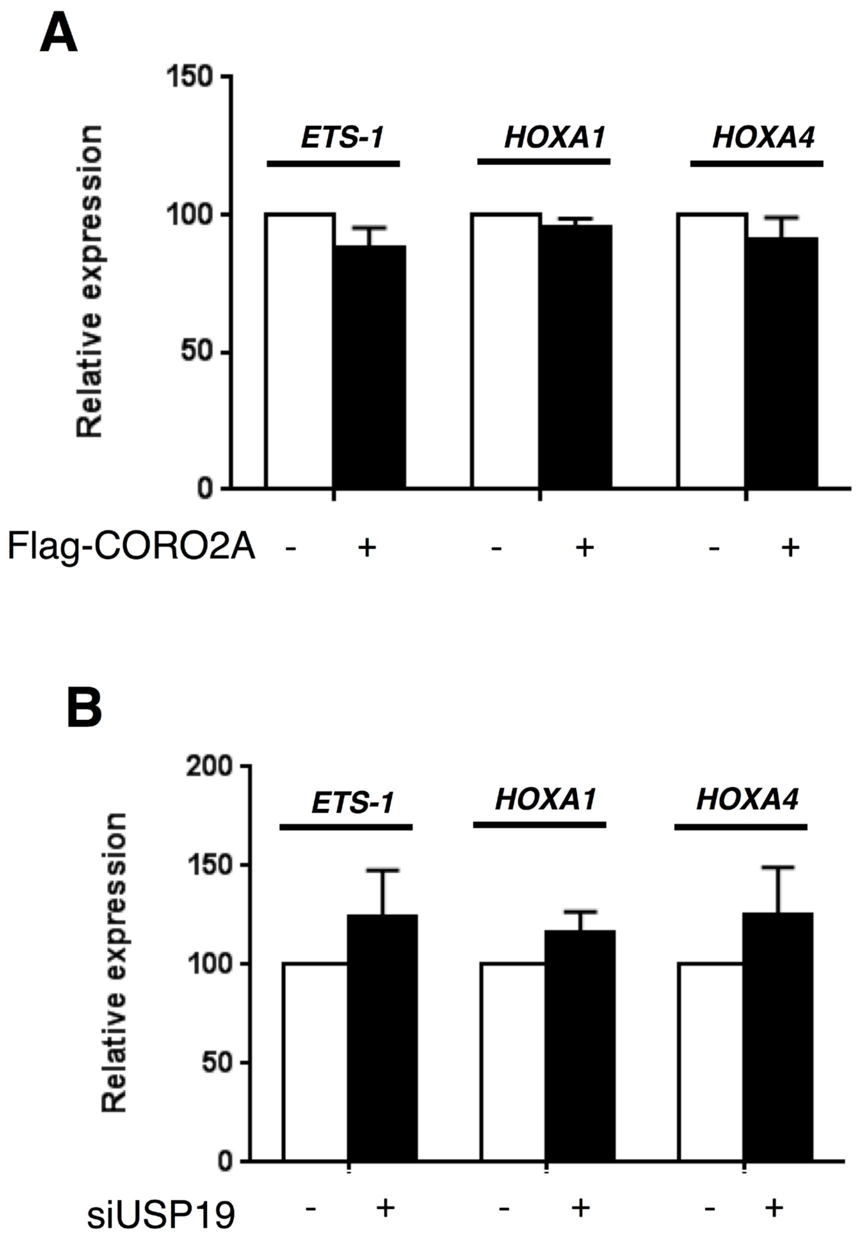

Supplementary Figure S2: The levels of transcription for *Ets-1*, *HOXA1*, and *HOXA4* in the expression of CORO2A and knockdown of USP19. RT-PCR was preformed with cDNA which obtained from **A.** Flag-tagged CORO2A or **B.** USP19 siRNA transfected MCF7 cells. *Ets-1, Hoxa1,* and *Hoxa4* primers were used for RT-PCR.
